# Design and experiments with a SLAM system for low-density canopy environments in greenhouses based on an improved Cartographer framework

**DOI:** 10.3389/fpls.2024.1276799

**Published:** 2024-01-29

**Authors:** Haoran Tan, Xueguan Zhao, Changyuan Zhai, Hao Fu, Liping Chen, Minli Yang

**Affiliations:** ^1^College of Engineering, China Agricultural University, Beijing, China; ^2^Intelligent Equipment Research Center, Beijing Academy of Agriculture and Forestry Sciences, Beijing, China; ^3^National Engineering Research Center for Information Technology in Agriculture, Beijing, China; ^4^Beijing PAIDE Science and Technology Development Co., Ltd, Beijing, China

**Keywords:** mobile robot, lidar, simultaneous localization and mapping (SLAM), greenhouse, perception

## Abstract

To address the problem that the low-density canopy of greenhouse crops affects the robustness and accuracy of simultaneous localization and mapping (SLAM) algorithms, a greenhouse map construction method for agricultural robots based on multiline LiDAR was investigated. Based on the Cartographer framework, this paper proposes a map construction and localization method based on spatial downsampling. Taking suspended tomato plants planted in greenhouses as the research object, an adaptive filtering point cloud projection (AF-PCP) SLAM algorithm was designed. Using a wheel odometer, 16-line LiDAR point cloud data based on adaptive vertical projections were linearly interpolated to construct a map and perform high-precision pose estimation in a greenhouse with a low-density canopy environment. Experiments were carried out in canopy environments with leaf area densities (LADs) of 2.945–5.301 m^2^/m^3^. The results showed that the AF-PCP SLAM algorithm increased the average mapping area of the crop rows by 155.7% compared with that of the Cartographer algorithm. The mean error and coefficient of variation of the crop row length were 0.019 m and 0.217%, respectively, which were 77.9% and 87.5% lower than those of the Cartographer algorithm. The average maximum void length was 0.124 m, which was 72.8% lower than that of the Cartographer algorithm. The localization experiments were carried out at speeds of 0.2 m/s, 0.4 m/s, and 0.6 m/s. The average relative localization errors at these speeds were respectively 0.026 m, 0.029 m, and 0.046 m, and the standard deviation was less than 0.06 m. Compared with that of the track deduction algorithm, the average localization error was reduced by 79.9% with the proposed algorithm. The results show that our proposed framework can map and localize robots with precision even in low-density canopy environments in greenhouses, demonstrating the satisfactory capability of the proposed approach and highlighting its promising applications in the autonomous navigation of agricultural robots.

## 1 Introduction

The development of agricultural facilities ensures the stable and safe supply of important agricultural products while reducing the occupation of arable land. These facilities are important for promoting the modernization of agriculture in rural areas (Bai et al., 2023). The development of intelligent equipment suitable for greenhouses is needed to ensure stable and efficient production in agricultural facilities (Jin et al., 2021; Zhai et al., 2022). Due to the narrow working environment of greenhouses, the applicability of conventional field operation equipment in greenhouses is limited. The traditional manual greenhouse management method has high labor intensity, and the application of pesticides in a closed environment may cause serious harm to the human body (Fu et al., 2022; Li et al., 2022). Therefore, it is necessary to study intelligent navigation robots that are suitable for application in greenhouse environments (Huang and Sugiyama, 2022; Qiao et al., 2022).

Environmental perception and real-time localization are the basis of greenhouse robot navigation studies and are prerequisites for autonomous navigation (Shamshiri et al., 2018; Chen et al., 2020). However, the closed nature of the greenhouse environment severely blocks satellite signals. Therefore, it is highly important to study perception and localization methods that do not depend on satellite navigation to develop navigation technology for greenhouse environments (Mendes et al., 2019; Choi et al., 2022). High-precision mapping and localization of agricultural robots are critical for the automation of greenhouse operations (Dong et al., 2020; Westling et al., 2021; Zhou et al., 2021). Using the prior map, the robot can obtain prior information about the environment and realize global path planning to achieve safe and accurate autonomous navigation. To this end, researchers have carried out many studies in the fields of sensor environment perception and simultaneous localization and mapping (SLAM) (Matsuzaki et al., 2018; Shi et al., 2020; Ouyang et al., 2022; Jiang and Ahamed, 2023; Su et al., 2023).

In recent years, with the development of computer technology and edge computing equipment, the sensors used for greenhouse operating environment perception and localization have included ultrasonic technology (Palleja and Landers, 2017; Chen et al., 2018; Lao et al., 2021), ultra-wideband (UWB) technology (Hou et al., 2020; Yao et al., 2021), LiDAR technology (Chen et al., 2019; Zhang et al., 2020; Saha et al., 2022; Sun et al., 2022), and machine vision technology (Nissimov et al., 2015; Wang et al., 2022a; Wang et al., 2022b). Huang et al. (2021) designed a robot localization system based on spread spectrum sounds for a greenhouse containing a strawberry ridge and achieved centimeter-level localization accuracy in a small greenhouse. However, the coverage of sound localization technology is limited, which significantly increases the cost in large-scale scenarios. Aiming to address the problem of insufficient features in greenhouse environments, Zhang et al. (2022) proposed a visual localization method based on benchmark markers and factor graphs. This method considers the constraint relationship between robot motion characteristics and variables, and the standard deviation of the localization error is less than 0.05 m. This method solves the problem of unstructured and insufficient features in greenhouses by adding benchmark markers to the SLAM front-end module. Although this method can achieve stable localization of the robot, it depends on the number of tags detected. In the case in which the plant is occluded or the number of tags is insufficient, inaccurate localization or even failure may occur. Yan et al. (2022) designed a loosely coupled real-time localization and mapping system based on an extended Kalman filter and visual-inertial odometry (VIO) using multisensor fusion and the visual-IMU-wheel odometry method to achieve accurate pose estimation and dense three-dimensional (3D) point cloud mapping in greenhouses. However, while the localization method based on visual SLAM performs well under good light conditions, the high light intensity in the environment interferes with the extraction of visual features, which may cause issues with map construction in the greenhouse environment and errors in robot pose calculation results.

To address the effects of greenhouse environment lighting on robot perception, LiDAR, which has advantages such as high stability and robustness, is a competitive perception and localization technology for all-weather greenhouse operations. The AgriEco Robot, designed by Abanay et al. (2022), is based on a two-dimensional (2D) LiDAR sensor and performs autonomous navigation. This robot can accurately navigate between rows of strawberry greenhouse crops, detect the end of a row, and switch to the next row. To solve the problem of inaccurate localization caused by the lack of structure in grape greenhouse scenes, Aguiar et al. (2022) designed a VineSLAM algorithm. The algorithm is based on 3D LiDAR point cloud extraction and uses half-plane features to construct an environmental map. The robot can achieve accurate localization in symmetrical long vineyard corridors. However, with seasonal changes, a decrease in grape canopy density affects map construction results and localization effects. Long et al. (2022) proposed a method to reduce the cumulative errors of odometers in long-distance greenhouse operations and the problem of mapping and localization accuracy. Their method is based on the UWB/IMU/ODOM/LiDAR-integrated localization system and integrates LiDAR with the two-dimensional map established through the adaptive Monte Carlo localization (AMCL) algorithm for global localization of the robot. The method is suitable for relatively open greenhouse environments where shorter crops are planted. However, in greenhouse environments with taller crops, due to the physical characteristics of UWB technology, the occlusion of plants affects the stability of the SLAM system. However, due to the large number of unstructured crops and irregular planting gaps in greenhouses, it is difficult for LiDAR-based methods to obtain clear map boundaries. Hou et al. (2020) developed a greenhouse robot navigation system based on a Cartographer with dual LiDAR. This system improved the efficiency of map building and enabled robotic mapping and autonomous navigation in a strawberry greenhouse environment. However, as the environmental map construction of strawberry plants is based on strawberry ridges, the results cannot be generalized to typical unridged greenhouse environments. At present, there are no researchers dedicated to solving the laser SLAM problem in greenhouse sparse feature environments. In the industrial field, Xie et al. (2021) proposed a visual-inertial fusion SLAM method for sparse lunar feature environments that integrates visual measurements and inertial sensor information via pose optimization methods to achieve high-precision joint positioning and improve the accuracy of relative pose estimation between key frames.

Although the above studies proposed improved methods to address problems such as greenhouse terrains, cumulative odometer errors, and irregular greenhouse structures, the impact of the sparse canopy characteristics of greenhouse crops on laser SLAM-based methods has not been considered. For crop environments such as low-density canopies in greenhouses, traditional laser SLAM methods have difficulty constructing accurate and complete environmental maps due to the loss of contour information caused by map degradation; thus, these methods cannot meet the localization accuracy needs for agricultural robot operation. In addition, SLAM methods based on 2D LiDAR can obtain environmental information only at the installation height level, while SLAM methods based on 3D LiDAR have higher computational costs and higher requirements for achieving good industrial computing performance (Jiang et al., 2022). The purpose of this study was to explore a low-cost spatial downsampling-based map construction and localization method based on the Cartographer framework to optimize the map construction effect and localization accuracy in suspended crop environments in greenhouses to construct maps and realize high-precision pose estimates in low-density canopy environments in greenhouses.

## 2 Materials and methods

### 2.1 Test platform

**Figure 1:**
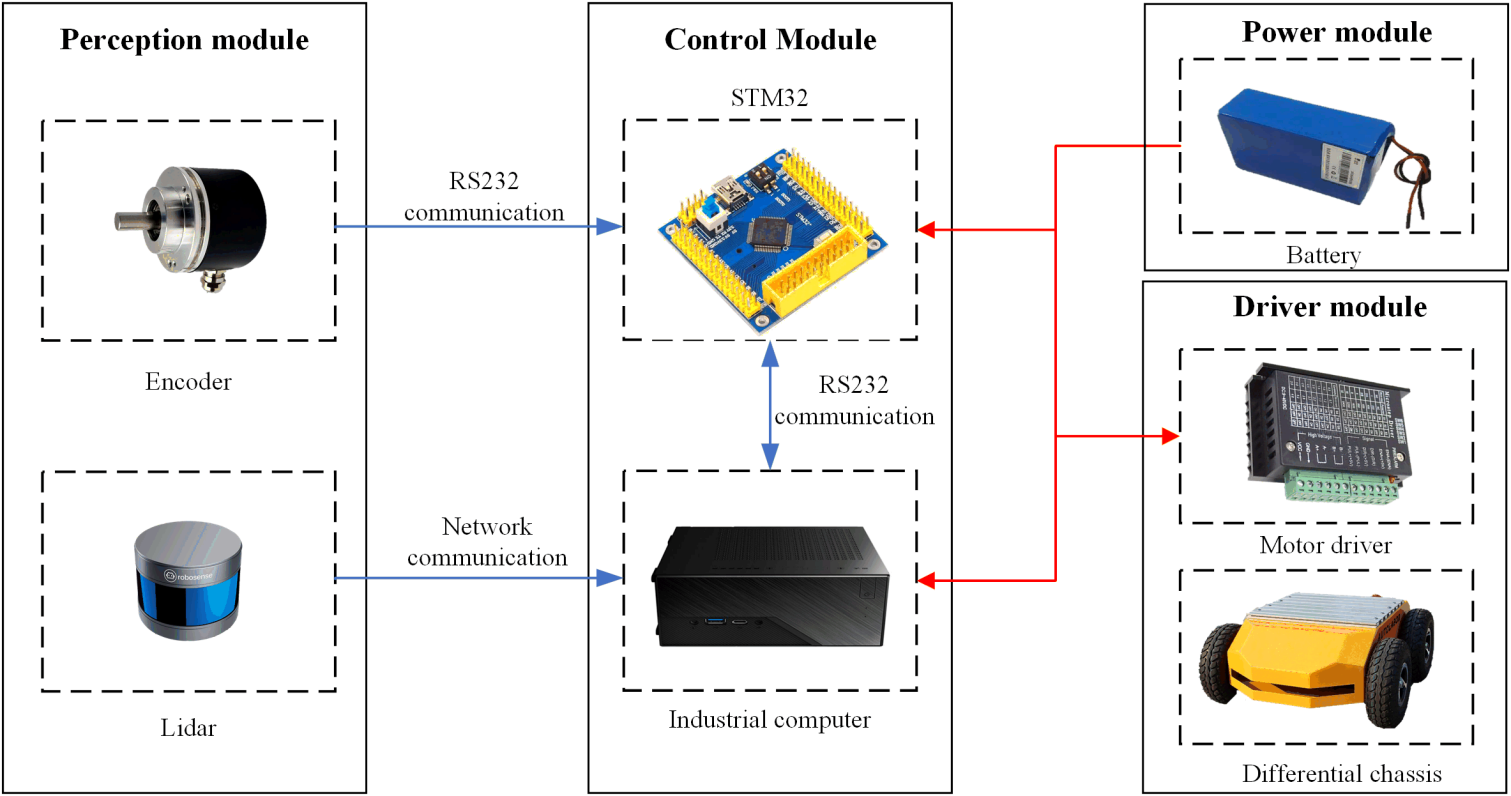
Map construction and localization hardware system of the greenhouse mobile robot.

The map construction and localization hardware system built in this paper is primarily composed of a perception module, control module, power module, and drive module, as shown in **Figure 1**. The Autolabor Pro1 platform produced by Qingke Intelligent Company (Shanghai, China) was selected as the robot chassis. The maximum movement speed of the robot is 1 m/s, and the robot can carry a maximum load of 50 kg. The four-wheel differential control of the robot can achieve stationary turning, allowing for turning in narrow spaces. The size was 726 × 617 × 273 mm, and the system was equipped with an RS-LiDAR-16 three-dimensional LiDAR system from Suteng Juchuang Company (Shenzhen, China). The LiDAR system can collect 300,000 data points per second, the acquisition frequency ranges from 5 to 20 Hz, the horizontal viewing angle range is 360°, and the resolution ranges from 0.1° to 0.4°. The vertical viewing angle is 30°, and the resolution is 2°. The measurement range is 0.2–100 m. The industrial computer that the robot is equipped with the Ubuntu 18.04 operating system, and the algorithm for the overall software system of the greenhouse mobile robot is designed based on the ROS Melodic. The specific model of the sensor is shown in **Table 1**.

**Table 1:** Greenhouse map construction and localization system equipment model.

No.	Equipment	Specification
1	Encoder	HTS-5008 encoder
2	LiDAR	RS-LiDAR-16LiDAR
3	Battery	24 V 40AH lithium iron phosphate battery
4	Motor drive	BLDH-750 brushless motor drive
5	Controller	STM32F103zet6 computer on a chip
6	Industrial computer	AMD Ryzen3 3200G
7	Chassis	Autolabor Pro1

The STM32 microcontroller can control the linear speed and angular velocity of the mobile robot, provide real-time feedback based on the motion state information of the robot, and interact with the industrial computer through RS232 serial communication. The industrial computer collects, fuses, and processes the robot sensor information; constructs the map; plans the path; and performs autonomous localization and navigation according to the control instructions. The ROS system installed on the industrial computer has a distributed architecture, which allows each functional module in the framework to be designed and compiled separately during runtime, with loose coupling between the modules.

### 2.2 Research method

#### 2.2.1 Improved Cartographer algorithm design

**Figure 2:**
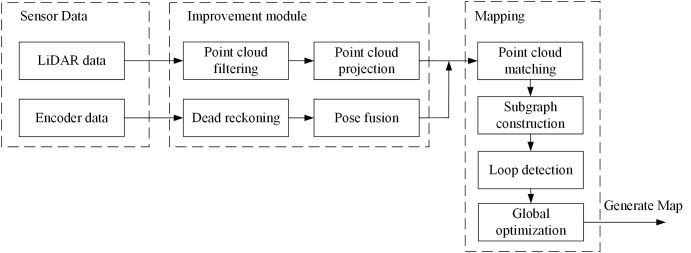
AF-PCP SLAM algorithm framework. AF-PCP, adaptive filtering point cloud projection.

The Cartographer algorithm (Hess et al., 2016) is an open-source laser SLAM framework proposed by Google that is widely used in the fields of robotics and autonomous systems. The Cartographer algorithm mainly establishes a series of submaps based on LiDAR data, inserts point cloud data into the submap through scan matching, and forms a complete map through loop closure detection and optimization, eliminating the cumulative error between submaps. The software framework of the adaptive filtering point cloud projection (AF-PCP) SLAM system based on the Cartographer algorithm is shown in **Figure 2**.

The LiDAR driver releases 3D point cloud data/rsldiar_points and performs *z*-axis threshold filtering based on the point cloud by setting the LiDAR perception region of interest (ROI). The key parameters of the algorithm are as follows: *min_height* represents the minimum height of the *z*-axis involved in point cloud compression, and the unit is m; *max_height* represents the maximum height involved in point cloud compression, and the unit is m; *range_min* and *range_min* represent the minimum and maximum measurement ranges of the output point cloud, respectively, and the units are both m; and *scan_time* represents the scanning time, and the unit is s. The data format of the input point cloud is sensor_msgs/PointCloud2, and the message format of the processed point cloud is sensor_msgs/LaserScan. The pose of the robot is estimated by a two-wheel differential kinematics model, and the odometer data are published in the message format of nav_msgs/Odometry.

**Figure 3:**
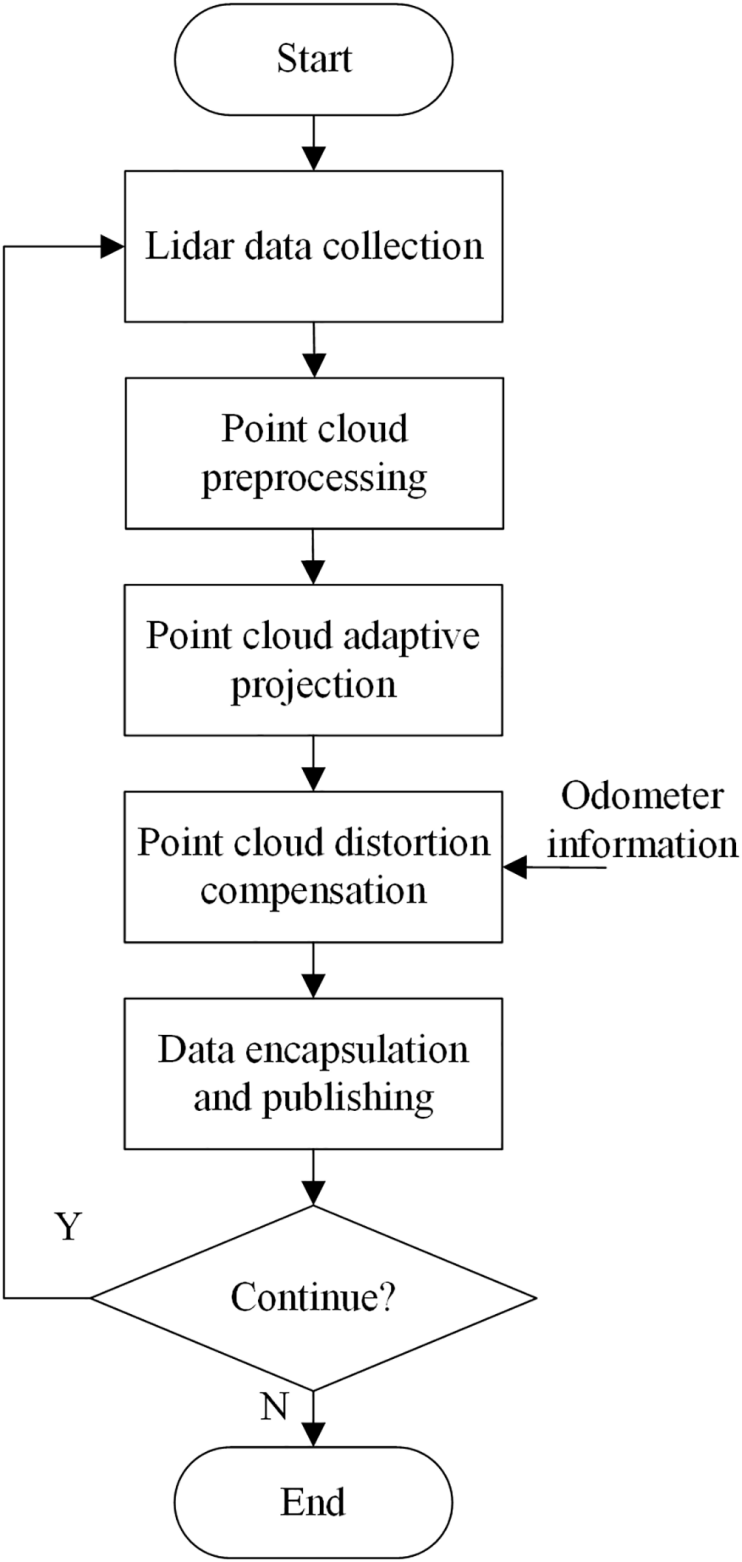
Flowchart of the greenhouse crop point cloud adaptive projection algorithm.

The greenhouse tomato plant is taken as an example, and the height of the tomato plant is 1.5–2 m. First, the ROI of the greenhouse robot is set, and the ROI is selected based on the LiDAR coordinate system to delineate a three-dimensional ROI. The motion speed of the greenhouse robot, the braking distance, and the reserved space between the body and the crops are considered in determining the area. The specific parameters are set as follows: *min_height* is set to 0.5 m, *max_height* is set to 1.5 m, *scan_time* is set to 0.1 s, *range_min* is set to 0.2 m, and *range_max* is set to 15 m. Based on the above parameter settings, the adaptive projection process for the greenhouse crop point clouds is designed, as shown in **Figure 3**.

After receiving a frame of complete multiline LiDAR data, the greenhouse map construction system filters the point cloud data outside the perceived ROI area. The filtered point cloud data $p=\{p_{1},p_{2},p_{3}\ldots p_{n}\}$ are stored in the array *points_range*. For the adaptive vertical projection of the point cloud, the specific processing steps are as follows: First, the *k*th scanning frame of the LiDAR $p_{k}$ is selected, each point in the frame is represented $p_{k}(x,y,z,i)$ , and an iterator is used to record the starting position of the point. The *x* coordinate information of the point cloud is obtained; the *y* and *z* information and intensity value *i* corresponding to the point are obtained using the offset pointers *offset_y*, *offset_z*, and *offset_i*; the point $p_{k}(x_{k},y_{k},z_{k},i_{k})$ is represented in the LiDAR Cartesian coordinate system. The points are converted to the polar coordinate representation of the *x*–*y* plane by vertical projection. The Euclidean distance $\rho_{k}$ , angle $\theta_{k}$ , and index $index_{k}$ of the point in the polar coordinate system are calculated, and the calculation formulas are shown in Formulas 1–4.


(1) $$p_{k}=\{p_{k},\theta_{k},i_{k}\}$$

(2) $$\rho_{k}=\sqrt{x_{k}^{2}+y_{k}^{2}}$$

(3) $$\theta_{k}=\arctan\frac{y_{k}}{x_{k}}$$

(4) $${\text{index}}_{k}=\frac{\text{arctan}\frac{y_{k}}{x_{k}}+\pi }{r}$$

where $\rho_{k}\;\theta_{k}$ are the distance and angle of the laser point in the polar coordinate system, $i_{k}$ is the corresponding intensity value, $x_{k}$ and $y_{k}$ are the corresponding coordinate values, and *r* is the angular resolution of the LiDAR system. The selected LiDAR angle beam contains 1,800 lines, and the angular resolution calculation formula is $r=\frac{2\pi }{1800}$ ≈ 0.00349.

Similarly, the point distances at this angle for the remaining 15-beam LiDAR data are calculated. The minimum distance obtained by the sorting algorithm is stored in the *scan_ranges* container as the distance value of the point.

Since the acquisition of laser point cloud data is not instantaneous, the robot distorts the motion of the point cloud data in the motion state. To eliminate this motion distortion, in this paper, the wheel odometer data calculated by the encoder are used to increase the pose update frequency, reflecting the pose change of the greenhouse robot during the laser data acquisition process. By calculating the odometer pose in the coordinate system corresponding to each point cloud in the current frame of the point cloud data, the coordinates of each point cloud are transformed into the same coordinate system, with the point of the first laser serving as the origin according to the obtained pose.

It is assumed that the robot accelerates uniformly in the process of collecting a frame of point cloud data. To ensure the accuracy of the data, a double-ended queue is used to save the point cloud data and ensure that there are at least two data points in the queue to prevent the data time of the wheel odometer from being less than the time of receiving the point cloud data. The start time and end time for a frame of point cloud data are $t_{start}$ and $t_{end}$ , respectively, and the corresponding starting position and end position of the origin of the LiDAR coordinate system in the odometer coordinate system are expressed as $p_{start}$ and $p_{end}$ , respectively. A total of *n* pose information data points $\left\{P_{start},\;P_{start+1},P_{start2,},\ldots ,P_{start+n-1},P_{end}\right\}$ are obtained. Linear interpolation is performed based on the LiDAR pose information to obtain the approximate odometer pose corresponding to the timestamp for the point cloud data. By transforming the point cloud data into the odometer coordinate system, LiDAR point cloud data based on the robot coordinate system can be obtained in cases with distortion. The method for calculating the coordinates of point $o_{x},o_{y}$ in the odometer coordinate system is shown in Formula 5:


(5) $$\left[\begin{array}{c} o_{x}\\ o_{y} \end{array}\right]=R\left[\begin{array}{c} x_{1}\\ y_{1} \end{array}\right]+l$$

(6a) $$R=\left[\begin{array}{cc} \cos\theta & -\sin\theta \\ \sin\theta & \cos\theta \end{array}\right]$$

(7a) $$l=\left[\begin{array}{c} x_{t}\\ y_{t} \end{array}\right]$$

where $\left(o_{x},o_{y}\right)$ are the coordinates of the odometer coordinate system after conversion, $\left(x_{1},y_{1}\right)$ are the point cloud data points before the transformation, and *R* (Formula 6a) is a rotation matrix that describes two coordinate systems. *l* (Formula 7a) is a translation vector that describes two coordinate systems, *θ* is the polar coordinate system angle corresponding to the point cloud, and $\left(x_{t},y_{t}\right)$ is the translation amount from the odometer coordinate system to the LiDAR coordinate system.

The laser point cloud data are transformed from the odometer coordinate system to the reference coordinate system of the data frame, and the starting point in the coordinate system of the current frame of the point cloud data is the reference coordinate system of the data frame. The method for calculating the coordinate of the transformed point $\left(o_{x}^{'},o_{y}^{'}\right)$ in the reference coordinate system is shown in Formula 6b:


(6b) $$\left[\begin{array}{c} o_{x}^{'}\\ o_{y}^{'} \end{array}\right]=R^{-1}\left[\begin{array}{c} o_{x}\\ o_{y} \end{array}\right]-R^{-1}\left[\begin{array}{c} x_{0}\\ y_{0} \end{array}\right]$$

where $\left(x_{0},y_{0}\right)$ are the coordinates of the origin of the reference coordinate system in the odometer coordinate system and *R^−^*^1^ is the inverse matrix that describes the rotation matrix of two coordinate systems.

**Figure 4:**
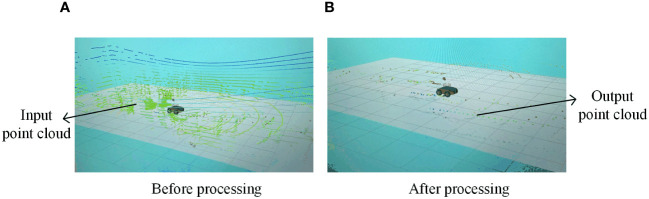
Point cloud processing results. **(A)** Before processing point cloud. **(B)** After processing point cloud.

Finally, the frame head timestamp, intensity value, and other information for each frame of the LiDAR point cloud data are supplemented, and the LiDAR data after the adaptive vertical projection process are output in the sensor_msgs/LaserScan format with a release frequency of 10 Hz. The effect of the point cloud processing is shown in **Figure 4**.

**Figure 5:**
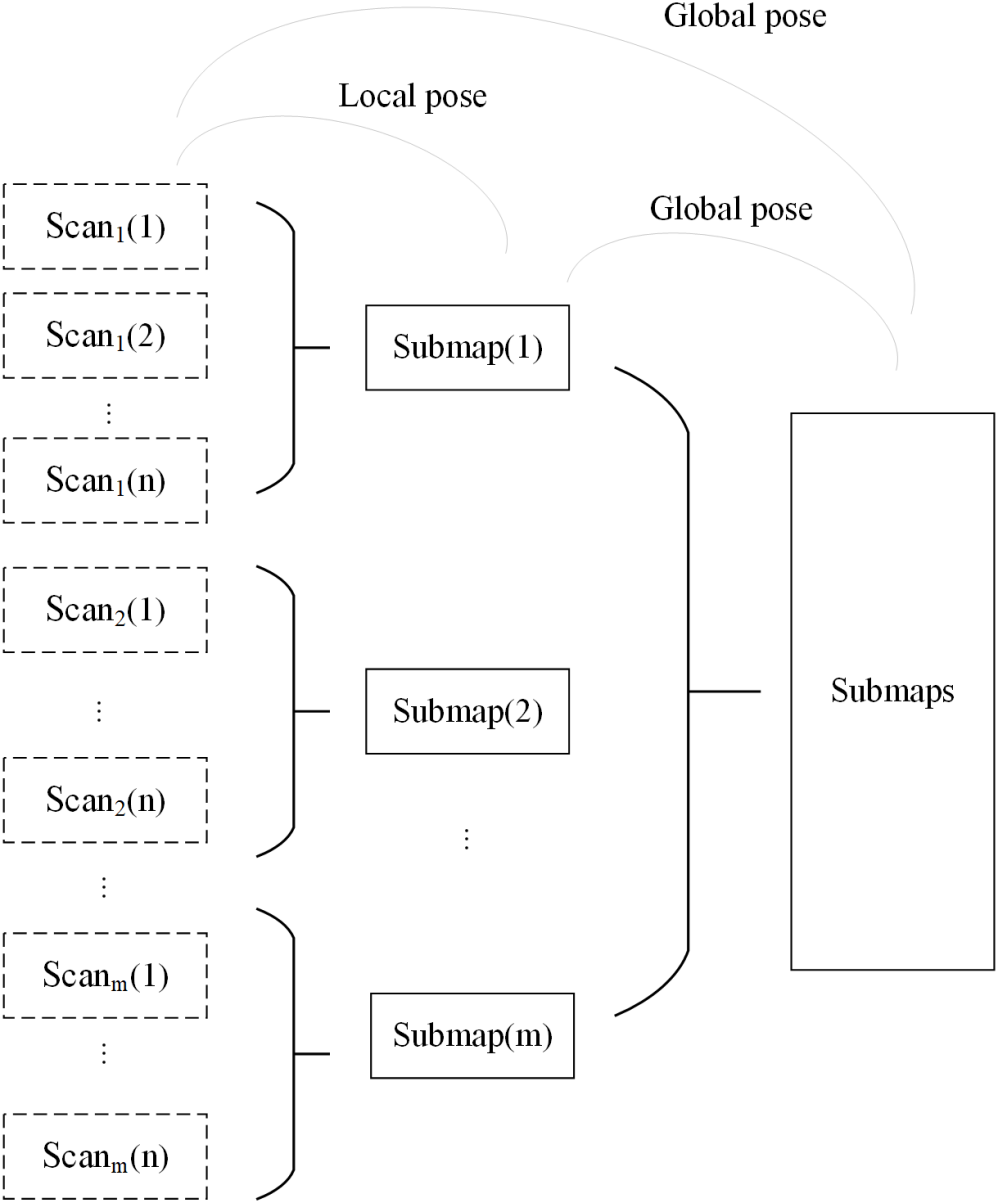
Schematic diagram of the map construction process.

The scan-to-map matching method was used to construct the map, as shown in **Figure 5**. First, we used a submap to organize the whole map. Each submap consists of several LiDAR scanning frames {Scan(n)}, and the complete global map is composed of all the submaps. Assuming that the initial pose of the robot is *ξ*_1_ = (0, 0, 0), the LiDAR scanning frame is denoted as Scan_1_ (1). At this pose, the first submap (1) is initialized by Scan_1_ (1). The robot pose *ξ*_2_ corresponding to Scan_1_ (2) is calculated by the scan-to-map matching method, and Scan_1_ (2) is added to Submap (1) based on pose *ξ*_2_. The scan-to-map matching method is continuously executed, and the newly obtained LiDAR frames are added until the new LiDAR frame is completely included in Submap (1); that is, when the new LiDAR frame does not contain new information other than that included in Submap (1), the creation of Submap (1) is completed. The above process is performed to create all the submaps (m). Finally, all the local subgraphs {Submap(m)} are used to form the complete global map.

#### 2.2.2 Layout of the test site

**Figure 6:**
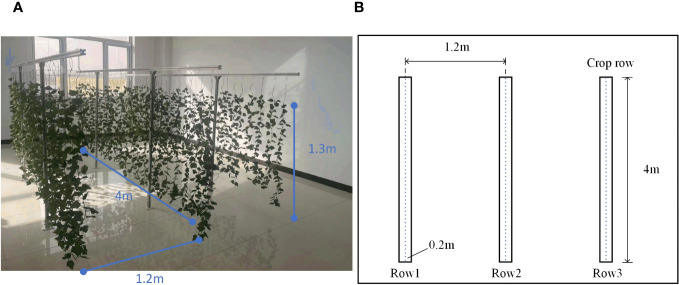
Simulated greenhouse test scene. **(A)** Physical display of simulated test scenarios. **(B)** Schematic diagram of simulation test scenario.

In this paper, a simulated greenhouse scene was built, which included 30 mm * 30 mm aluminum profiles, fixed bases, and simulated plants. The simulated crop has three rows; each row is 4 m long, the row height is 1.3 m, and the row spacing is 1.2 m. The test scene is shown in **Figure 6**. In the crop row, aluminum profiles were used as support rods every other distance, a set of simulated crops was arranged every 0.2 m, and the number of leaves per crop was counted.

### 2.3 Test scheme

#### 2.3.1 Different sparse degree mapping tests

To verify the mapping effect of the algorithm for different sparse canopy crops in the greenhouse, based on the robot platform built in Section 2.1 and the simulated greenhouse scene constructed in Section 2.2.2, the crops were randomly pruned five times, and 10% of the leaves were pruned each time. A total of six simulated environments with different degrees of sparseness were generated. The experiment was carried out at the National Agricultural Information Demonstration Test Base in Xiaotangshan town, Beijing, in May 2023. In this experiment, the AF-PCP SLAM algorithm was used for SLAM mapping, and the Cartographer algorithm was used for the control group. The remote control robot moves between the crop rows to construct a map of the entire environment. The specific mapping operation was performed as follows:

1 The ROS core node, chassis control node, and LiDAR mapping node are started.
2 The robot movement speed is set to 0.2 m/s, and the remote control operation is performed according to the running track for the interrow operation.
3 The visualization tool Rviz is used to monitor the robot mapping results in real time, and the map is saved after map construction is completed.

The canopy biomass, leaf area (LA), leaf area index (LAI), and leaf area density (LAD) are the main indicators of canopy density. The distribution of leaf density in the canopy is random and unpredictable in three-dimensional space, which complicates the quantitative analysis process (Gu et al., 2021). In this paper, the LAD was used as a measure of canopy density to divide the six experiments. A YMJ-G leaf area meter (Shandong Fangke Instrument Co., Ltd., Shandong, China) was used to measure the leaf density 10 times. The average leaf area was 21.203 cm^2^, and environments with six LAD values were constructed. The number of crop row pixels, the crop row length, and the maximum gap length for each LAD were used as evaluation indicators. The actual length of the crop row was 3.985 m, and the actual value of the crop row spacing gap was 0.08 m. The method for counting the number of pixels involved counting the number of pixels in the middle crop row in the PGM image via the two algorithms. The crop row length and maximum gap length were calculated by importing the Pbstream file calculated by the two algorithms into the cost map tool under the ROS open source function package Movebase; the scale measurement tool in Rviz was used to measure the crop row length in the middle row, and the maximum gap length was determined.

#### 2.3.2 Localization system performance test

**Figure 7:**
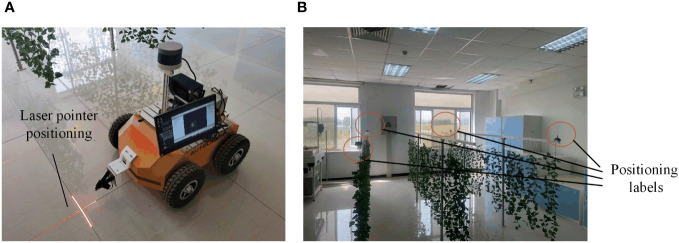
Pose alignment and test environment localization equipment construction. **(A)** Using a laser pointer for pose alignment. **(B)** test environment localization equipment construction.

To verify the localization accuracy of the proposed method at different speeds, the robot was controlled to move along the crop row by setting different robot motion speeds, namely, a low speed of 0.2 m/s, a medium speed of 0.4 m/s, and a high speed of 0.6 m/s. The ROSBag tool was used to record the real-time data of the sensor and the localization algorithm output results. The starting position in each test was aligned with a laser pen to ensure that the initial pose in each test was the same, as shown in **Figure 7A**.

In this paper, according to the localization evaluation equipment selected in Reference (Zhang et al., 2022), the Starter Set Super-MP-3D ultrasonic localization device from the Marvelmind Company was selected to determine the true value to evaluate the localization accuracy of the robot. The device consists of four fixed labels, a mobile vehicle label, and a localization route. The device can theoretically obtain an accuracy of up to ±2 cm. In addition, in this paper, the dead reckoning localization method was used as a comparison method to evaluate the performance of the AF-PCP SLAM algorithm comprehensively. Four localization labels were strategically placed in the greenhouse, and four vertices in the test area were selected for placement. To reduce the interference of crop occlusion on ultrasonic signals and ensure maximum signal coverage, four positioning labels were placed at the vertices of two outer crop rows. Fixing labels at the same height can improve positioning accuracy. Each label was fixed on a beam 1.4 m above the ground, as shown in **Figure 7B**.

To prove the effectiveness of the AF-PCP SLAM algorithm, the localization accuracy was evaluated using EVO (https://github.com/MichaelGrupp/evo, accessed on June 28, 2022). To verify the results, the ultrasonic localization data were used as the ground truth to analyze the localization effect. The relative pose error (RPE) describes the accuracy of the two-frame pose difference between the estimated pose and the real pose at a fixed time difference *t*, which is equivalent to the error of directly measuring the pose results. The RPE for frame *i* is shown in Formula 7b:


(7b) $$E_{\text{i}}={\left(Q_{i}^{-1}Q_{i+\Delta t}\right)}^{-1}(P_{i}^{-1}P_{i+\Delta t})$$

where $E_{\text{i}}$ represents the RPE of the *i*th frame, $Q_{i}$ represents the true ultrasonic pose value, $P_{i}$ represents the estimated pose value, and Δt represents a fixed interval time coefficient.

Assuming that there are *n* pose frames, the *n* − Δt RPE values can be calculated, and the total value is obtained using the root mean square error (RMSE) statistics, as shown in Formula 8:


(8) $$RMSE(E_{1:n},\Delta t)={\left(\frac{1}{m}\sum_{i=1}^{m}\|trans(E_{i})\|2\right)}^{\frac{1}{2}}$$

Here, m=n−Δt and $trans(E_{i})$ represent the translation of the RPE. To comprehensively evaluate the performance of the algorithm, the average RMSE is calculated, as shown in Formula 9:


(9) $$RMSE(E_{1:n})=\frac{1}{n}\sum_{\Delta t=1}^{n}RMSE(E_{1:n},\Delta t)$$

## 3 Results

### 3.1 Mapping performance

**Figure 8:**
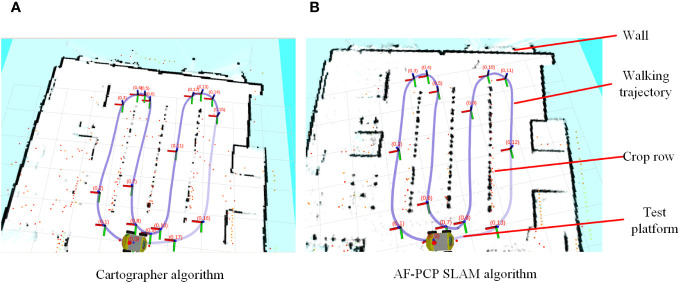
Visualization of the results of the Rviz map construction and localization trajectories. **(A)** The map construction results of the Cartographer algorithm. **(B)** The map construction results of the AF-PCP SLAM algorithm.

To verify the wide applicability of the AF-PCP SLAM algorithm for different crop densities, six tests were carried out in the same environment. The localization trajectory and surrounding environment information of the different methods are displayed in real time in Rviz, as shown in **Figure 8**.

**Figure 9:**
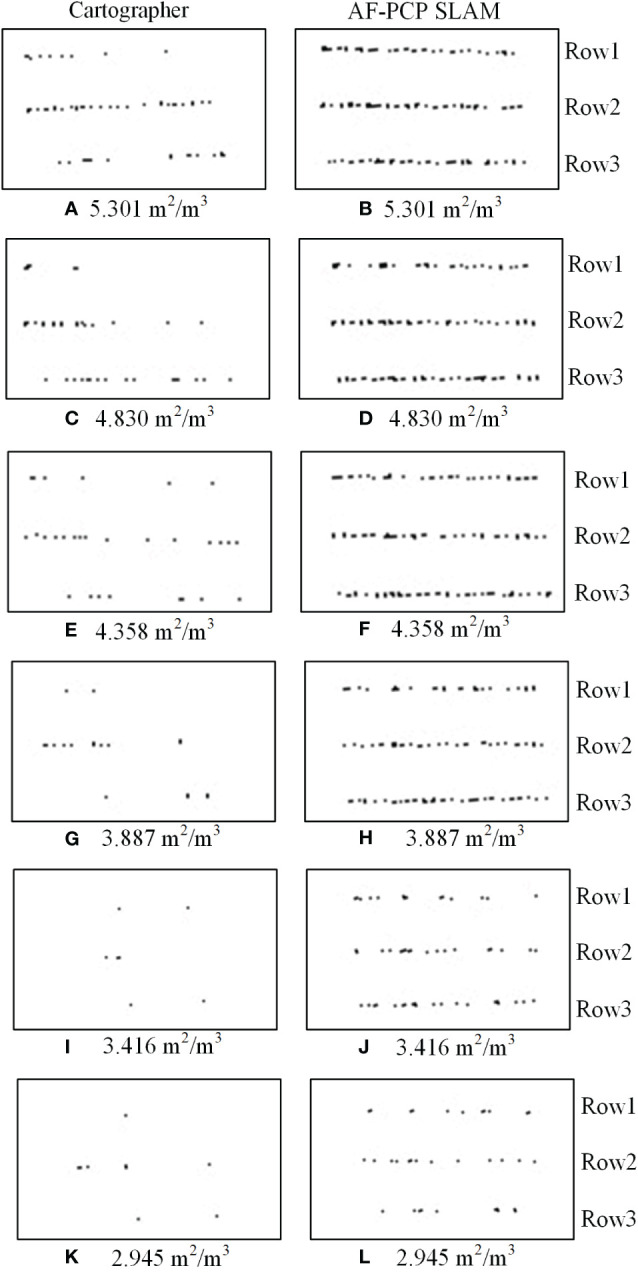
Results of the mapping of the two algorithms. The map construction results of the Cartographer algorithm under sparsities of **(A)** 100%, **(C)** 90%, **(E)** 80%, **(G)** 70%, **(I)** 60%, and **(K)** 50%. The map construction results of the AF-PCP SLAM algorithm under sparsities of **(B)** 100%, **(D)** 90%, **(F)** 80%, **(H)** 70%, **(J)** 60%, and **(L)** 50%. AF-PCP, adaptive filtering point cloud projection.

**Figure 8** shows that the Cartographer algorithm accurately constructs structured walls and glass into grayscale grid maps. However, these maps are still not perfect. Unstructured suspended crops occupy most of the space in the greenhouse environment. However, based on the mapping results, the Cartographer algorithm results in a large loss of crop row mapping, whereas the AF-PCP SLAM algorithm yields more accurate mapping results. To better show the effect of the AF-PCP SLAM algorithm on crop row mapping under different degrees of sparseness, **Figure 9** shows the map construction results of the two methods for six degrees of sparseness.

**Figure 10:**
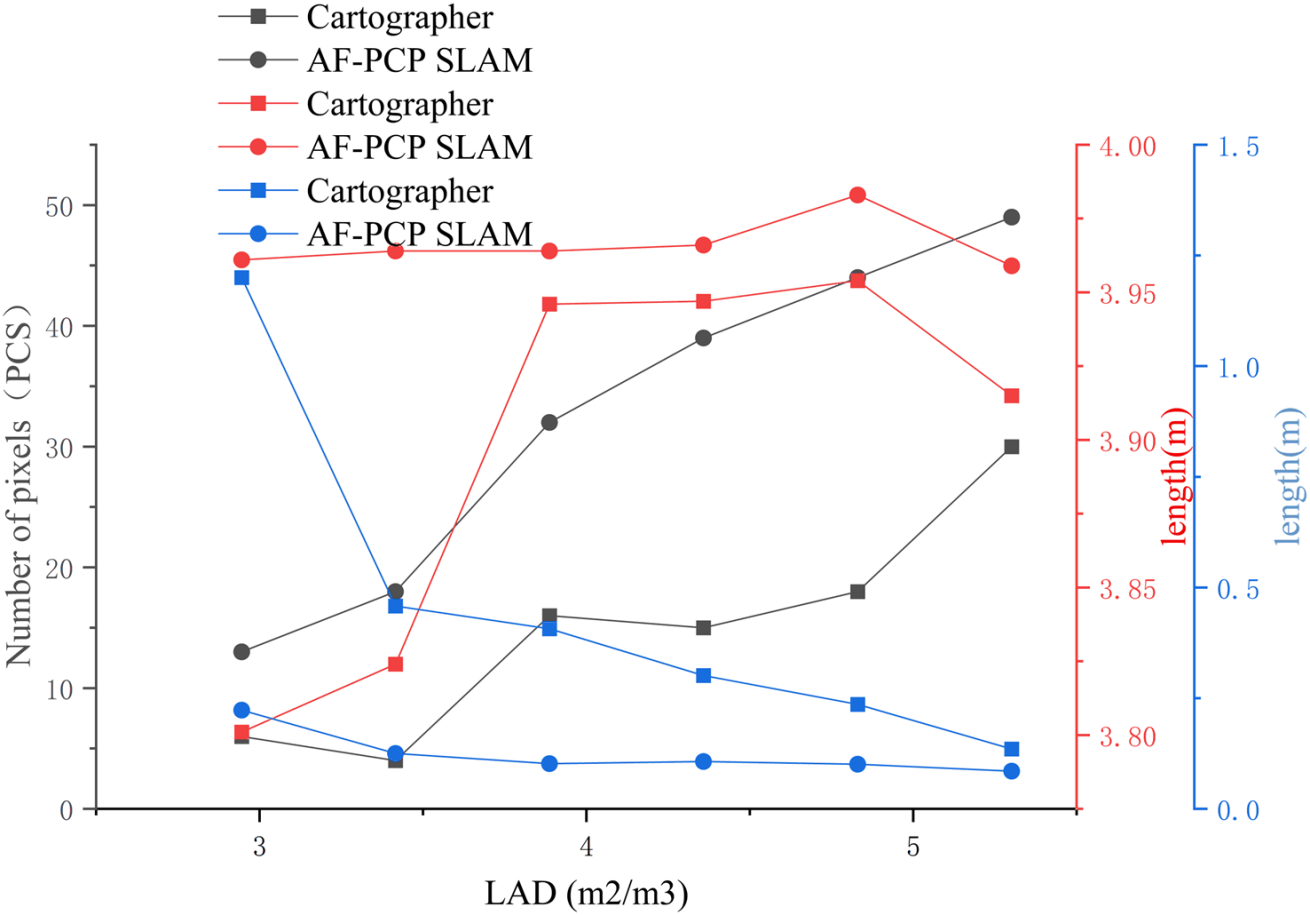
Map quantitative indicators under different LADs. The black, red, and blue symbols represent the number of pixels in the crop row, the length of the crop row, and the maximum gap length, respectively. LADs, leaf area densities.

**Figure 9** shows the crop mapping results of the Cartographer algorithm and AF-PCP SLAM algorithm for six degrees of sparseness. According to the LAD calculation method presented in Section 2.3.1, the LADs corresponding to the six sparsities are 5.301 m^2^/m^3^, 4.830 m^2^/m^3^, 4.358 m^2^/m^3^, 3.887 m^2^/m^3^, 3.416 m^2^/m^3^, and 2.945 m^2^/m^3^. The quantitative statistical results, including the number of pixels, the crop row lengths, and the maximum gap lengths, for the six LADs were calculated, as shown in **Figure 10**.

According to the results shown in **Figure 10**, as the LAD increased, the number of pixels in the crop row generally increased. The AF-PCP SLAM algorithm outperforms the Cartographer algorithm in terms of the number of pixels. The number of pixels in the crop row reflects the effective area of the grid map, and the results indicate that the AF-PCP SLAM algorithm is better at constructing the map of the crop row. Specifically, the number of pixels in the AF-PCP SLAM algorithm increased by 63.3%, 144.4%, 160%, 100%, 350%, and 116.7%, with an average increase of 155.7%.

In addition, as the LAD increased, the length of the crop rows constructed by the AF-PCP SLAM algorithm was close to the real length of the crop rows. When the LAD was greater than 3.877 m^2^/m^3^, the length of the crop row constructed by the Cartographer algorithm was close to the real length of the crop row. However, when the LAD was less than 3.877 m^2^/m^3^, the error between the crop row length constructed by the Cartographer algorithm and the real length was large. The average error in the crop row length calculated by the Cartographer algorithm was 0.086 m, and the coefficient of variation was 1.741%. The mean error of the crop row length constructed by the AF-PCP SLAM algorithm was 0.019 m, and the coefficient of variation was 0.217%. Thus, the mean error and coefficient of variation of the crop row length were reduced by 77.9% and 87.5%, respectively, with the AF-PCP SLAM algorithm.

Moreover, as the LAD increases, the maximum gap length decreases for both algorithms. When the LAD was 5.301 m^2^/m^3^, the maximum gap lengths obtained by the two methods were consistent with the true value of the set crop row spacing gap. When the LAD was between 3.887 m^2^/m^3^ and 4.83 m^2^/m^3^, the maximum gap length obtained by the Cartographer algorithm changed more slowly. However, when the LAD was between 2.945 m^2^/m^3^ and 3.416 m^2^/m^3^, the maximum gap lengths obtained by the Cartographer algorithm were 0.458 m and 1.2 m, respectively. The maximum gap lengths obtained by the AF-PCP SLAM algorithm were 0.125 m and 0.223 m, which were better than those obtained by the Cartographer algorithm. The maximum gap length obtained by the Cartographer algorithm was 1.2 m, and the mean value was 0.456 m. The maximum gap length obtained by the AF-PCP SLAM algorithm was 0.223 m, and the mean value was 0.124 m, which is 72.8% lower than that of the Cartographer algorithm.

The crop row length in the mapping results reflects the quality of the crop row end map construction to a certain extent, and the lack of a constructed row end map and the increase in the maximum gap length increase the error rate of the path planning algorithm. When the maximum gap length exceeds the width of the robot body, the path planning algorithm chooses a closer route, resulting in errors in the global path planning of the robot. When the LAD is reduced to 3.877 m^2^/m^3^, the mapping results of the Cartographer algorithm no longer represent most of the crop information in the environment, while the AF-PCP SLAM algorithm can still achieve accurate mapping of crop rows; thus, the AF-PCP SLAM algorithm has higher mapping robustness.

### 3.2 Localization performance

**Figure 11:**
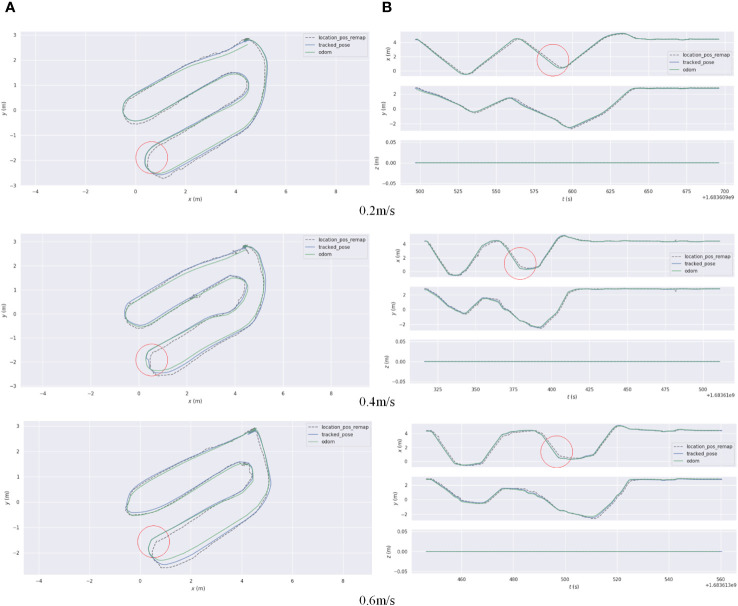
**(A)** A comparison of the absolute localization errors between the AF-PCP SLAM algorithm and Odom and the real trajectory. **(B)** A comparison of the error curves in the *x* and *y* directions over time. AF-PCP, adaptive filtering point cloud projection.

**Figure 12:**
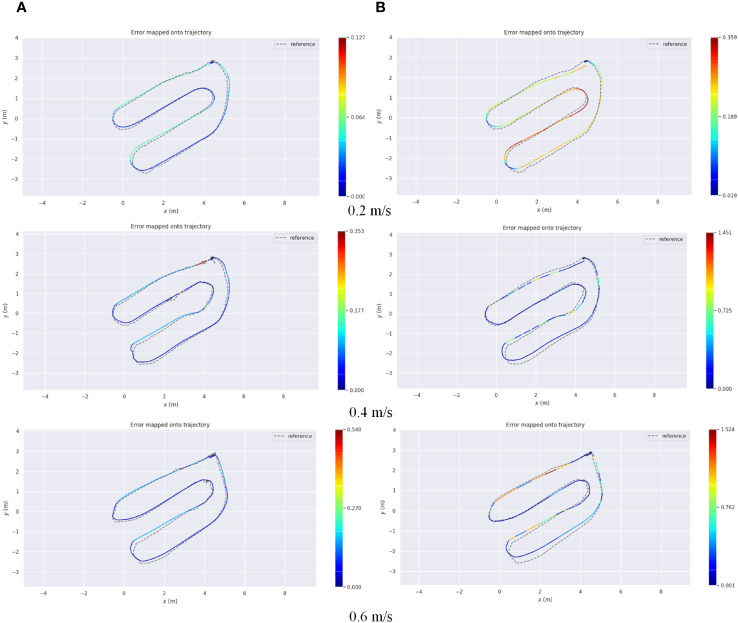
AF-PCP SLAM algorithm and Odom localization error. **(A)** The error in the trajectory of the AF-PCP SLAM algorithm. **(B)** The error in Odom. AF-PCP, adaptive filtering point cloud projection.

**Figure 13:**
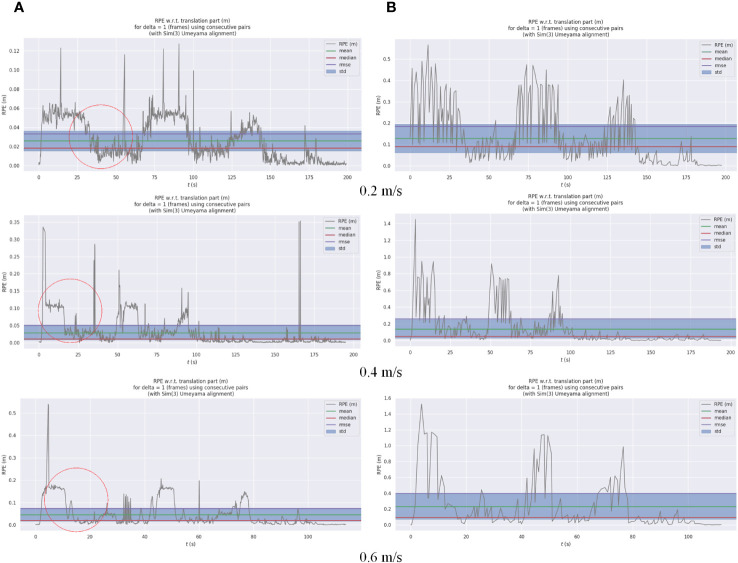
Odom and AF-PCP SLAM algorithm localization errors. **(A)** The curve of the relative pose error (RPE) of the AF-PCP SLAM algorithm with time. **(B)** The curve of the RPE of Odom with time. AF-PCP, adaptive filtering point cloud projection.

According to the localization accuracy evaluation criteria presented in Section 2.3.2, the localization errors of the AF-PCP SLAM algorithm and track deduction algorithm were calculated at different speeds. **Figure 11** shows the trajectories of robots operating at speeds of 0.2 m/s, 0.4 m/s, and 0.6 m/s and the error curves in the *x*, *y*, and *z* directions. The RPE was used to analyze these results, and for each timestamp, the absolute difference between the true pose and the estimated pose was calculated. To highlight the RPE during the robot’s movement, **Figure 12** shows the error between the AF-PCP SLAM algorithm, the track deduction algorithm, and the real trajectory and maps the error to the trajectory through color coding. **Figure 13** shows the curves of the RPE, mean, median, root mean square error, and standard deviation over time. The quantitative results of the error calculation are shown in **Table 2**.

**Table 2:** RPE values of the AF-PCP SLAM algorithm, Cartographer algorithm, and Odom.

Test ID	Methods	Max	Mean	Median	RMSE	Std
0.2	AF-PCP SLAM Cartographer Odom	0.127 0.371 0.567	0.026 0.038 0.129	0.018 0.039 0.090	0.033 0.063 0.185	0.021 0.022 0.133
0.4	AF-PCP SLAM Cartographer Odom	0.353 0.109 1.451	0.029 0.047 0.138	0.012 0.044 0.050	0.051 0.085 0.259	0.042 0.028 0.220
0.6	AF-PCP SLAM Cartographer Odom	0.540 0.842 1.524	0.046 0.035 0.233	0.020 0.031 0.095	0.074 0.087 0.397	0.058 0.020 0.322

RPE, relative pose error; AF-PCP, adaptive filtering point cloud projection; RMSE, root mean square error.

**Figure 11A** shows different trajectories: location_pos_remap represents the localization trajectory of the true ultrasonic value, tracked_pose represents the pose trajectory calculated by the AF-PCP SLAM algorithm, and Odom represents the pose trajectory calculated by the track inference algorithm. **Figure 11B** shows the time-varying pose values in the *x*, *y*, and *z* directions. Since the robot moves in a two-dimensional plane, the value in the *z* direction is always 0. In each experiment, three extreme values are generated, which represent the moment when the robot turns at the end of a row. The results of the three experiments show that the pose trajectory calculated by the AF-PCP SLAM algorithm is closer to the real value than that calculated by the track deduction algorithm. The red circle in **Figure 11** marks the position where the robot turns at the end of the last row, which corresponds to the maximum localization error.

According to the test results shown in **Figures 12**, **13**, the average localization error is 0.026 m, and the maximum localization error is 0.127 m when the robot moves at 0.2 m/s. When the robot moves at 0.4 m/s and 0.6 m/s, the initial fluctuation in the ultrasonic signal affects the maximum localization error; the average localization errors are 0.029 m and 0.046 m, respectively, which indicates that the localization accuracy is relatively stable. In contrast, the track deduction algorithm has a serious error accumulation problem. The average localization error of the track deduction algorithm is generally greater than 0.12 m, and the average localization error reaches as high as 0.233 m at a speed of 0.6 m/s. The AF-PCP SLAM algorithm is based on the localization of laser matching, which is not sensitive to error accumulation or the environment, so it has higher localization accuracy. The experimental results in **Table 2** show that the AF-PCP SLAM algorithm proposed in this paper can achieve high-precision localization of robots at movement speeds of 0.2 m/s, 0.4 m/s, and 0.6 m/s. The average localization error of the AF-PCP SLAM algorithm is reduced by 79.8%, 78.9%, and 80.3% at these three speeds compared with the error of the track deduction algorithm, and the average localization error is reduced by 79.9%.

### 3.3 The actual greenhouse environment performance

**Figure 14:**
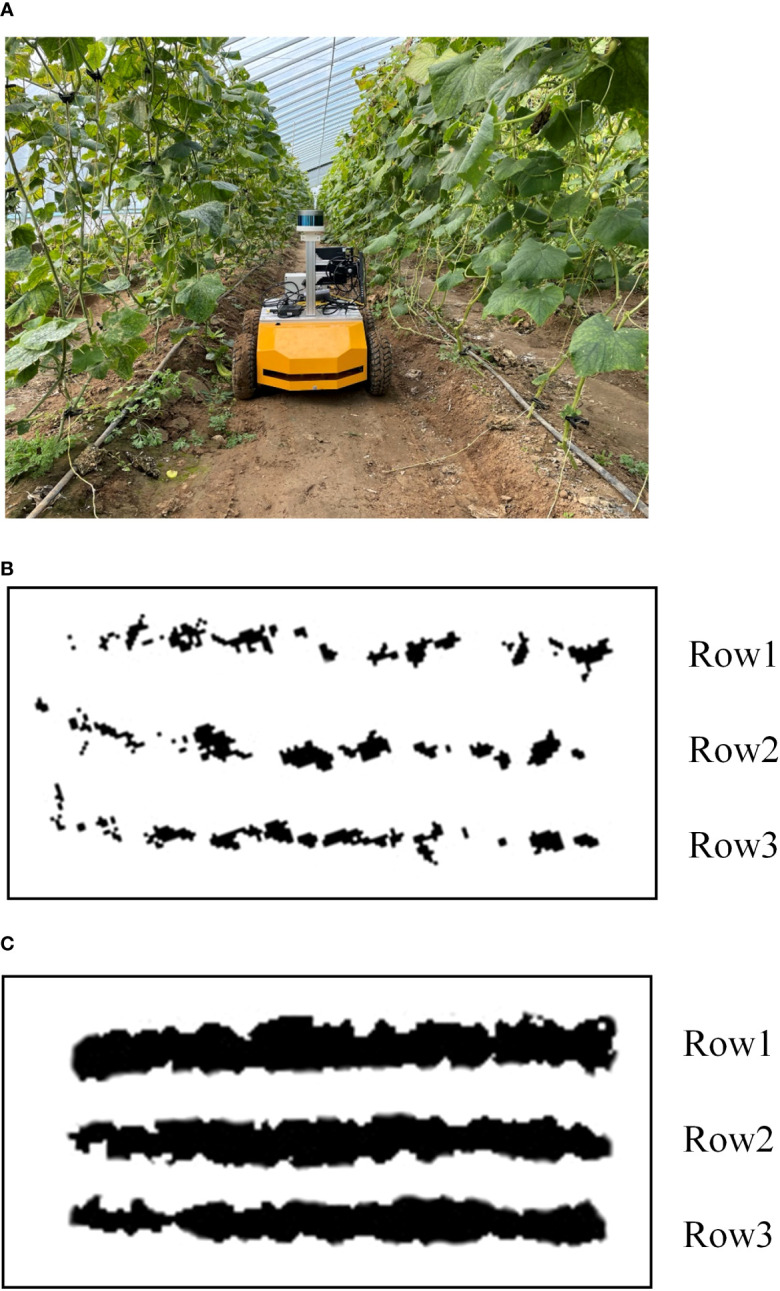
The actual greenhouse scenario test **(A)** The greenhouse test scenario. **(B)** The map construction results of the Cartographer algorithm. **(C)** Tthe map construction results of the AF-PCP SLAM algorithm.

To verify the ability of the AF-PCP SLAM algorithm to construct maps in actual greenhouse scenes, a greenhouse experiment was carried out at the “Doctor” farm base in Pinggu District, Beijing. The cucumber cultivar Yutian 156 was planted in this field, with a row width of 1.2 m. In the experiment, the Cartographer algorithm and AF-PCP SLAM algorithm were used to construct the map of the greenhouse. The robot walked along the crop rows at a speed of 0.2 m/s, and a map of the three rows of crops was constructed. The experimental environment and the map construction results of the two algorithms are shown in **Figure 14**.

**Figure 14A** shows the greenhouse test scenario, and **Figures 14B, C** are the map construction results of the Cartographer algorithm and AF-PCP SLAM algorithm, respectively. **Figure 14A** shows that due to the larger leaves of the actual greenhouse crops, the difference between the two algorithms in the actual greenhouse scene is more obvious. **Figures 14B, C** show that, compared with the Cartographer algorithm, the AF-PCP SLAM algorithm has a larger mapping area for crop rows. Accurate mapping of crop row contours can effectively control the moving trajectory and range of motion during robot navigation and effectively avoid damaging crops during robot operation. Moreover, row2 and row3 in **Figure 14B** exhibit obvious drifts because uneven ground reduces the mapping accuracy of crop rows and leads to map drift compared with that in the laboratory scene. The corresponding row2 and row3 drifts in **Figure 14C** are small, which also reflects, to some extent, that the AF-PCP SLAM algorithm has higher mapping robustness in actual greenhouse scenes.

## 4 Discussion

### 4.1 Discussion of the mapping results

Suspended plant patterns are widely used in greenhouses and agricultural environments. In this experiment, mobile robots walked along crop rows in greenhouses. However, the robot is in a repeated scene when walking between crop rows. Because crop rows usually have highly repetitive structures, with long, narrow, and tall rows, this poses a challenge in map construction. When constructing a map in a low-density canopy environment, crop rows may have missing content, such as voids and gaps. There are two reasons for this. First, when the laser beam passes through a gap in the leaves, another row of crops may be detected, or the wall may be directly detected, resulting in inconsistent observation results. This reduces the probability of obstacles in the occupied grid map corresponding to this position, which leads to the degradation of the map. Second, the irregular structure of the blade may cause the laser detection results of blades at the same position to be inconsistent under different robot poses. As the canopy leaf area density decreases, this situation is aggravated, resulting in the absence of crop rows on the map. To address these problems, in this paper, the innovative AF-PCP SLAM algorithm is designed, which fully considers the spatial characteristics of suspended crops in greenhouses, extracts and compresses the map contour based on 16-line LiDAR data, and maps the data to a 2D plane to establish the environment map and localize the robot. This innovative approach addresses the abovementioned map degradation problem, thereby improving the accuracy and robustness of the mapping results.

### 4.2 Discussion of the localization results

Due to the equidistant distribution of greenhouse crop rows, greenhouse environments have a high degree of symmetry, forming a “corridor scene”. The corridor problem is one of the key problems faced by the SLAM method. Because the LiDAR detection results are similar, they cannot reflect the actual displacement in the forward direction, which may introduce localization inaccuracies in the forward direction with the SLAM method. According to **Figure 11A**, in this experiment, the results estimated by the Odom localization method are accurate in the initial row. However, when the robot moves to the end of the crop row and turns into the next row, the localization error of the Odom algorithm in the *y* direction increases with increasing distance. When the robot returns to the starting point, the pose estimation error of the Odom algorithm is approximately 0.2 m. According to **Figure 11B**, the position of the extreme point corresponds to the trajectory in **Figure 11A**. The trajectory position of the red circle in **Figure 11A** corresponds to the error at the position of the red circle labeled in **Figure 11B**. The three tests show that the error in the *x* direction is the largest at this time. We believe that this phenomenon occurs because when the agricultural robot turns, the deviation in the *y* direction increases due to errors in the robot’s coordinate system. This is due to the limitations of the two-wheel differential model. Even after the odometer calibration, the deviation in the *y* direction increases rapidly as the distance increases. The *x*-direction error in **Figure 11B** is mainly caused by the *y*-direction error component in the robot coordinate system, so the maximum error is generated after the robot turns down the last row. At this moment, the localization error of the AF-PCP SLAM algorithm proposed in this paper is much smaller than that of the track deduction algorithm, which allows the robot to accurately navigate to the next crop row according to the path planning algorithm, thus reducing the risk of row-end collisions during robot navigation. The results shown in **Table 2** intuitively reflect the influence of the three moving speeds on the localization accuracy. With increasing moving speed, the mean, maximum, and standard deviation of the localization error increase. The reason is that the increase in the mechanical vibration of the robot causes additional input noise, which affects the collection of the point cloud data and the matching effect of the point cloud. Moreover, as the processing efficiency of the algorithm remains unchanged, an increase in the rate of change in the robot pose leads to a decrease in the localization accuracy.

In **Figure 12**, there is a certain regularity between the fluctuations in the RPE value and the extreme point position of the error curve in the *x* direction in **Figure 10B**. For example, the RPE value at the position indicated by the red circle in **Figure 12A** decreases in all three speed tests. The position of the decrease corresponds to the extreme point in the error curve, that is, the moment when the robot moves to the end of the row. Although the increase in the error is small (only 2 cm), there is a certain regularity in the results. This phenomenon may be related to robot system errors and may also be related to ultrasonic localization labeling errors. Through repeated comparisons, we believe that this phenomenon is related to the physical characteristics of the ultrasonic localization labeling method. When the robot moves between rows, the localization accuracy of the ultrasonic label is reduced due to the occlusion of the canopy crop. When the robot moves to the end of the crop row, the occlusion effect of the crop row is reduced. At this time, the vehicle label carried by the robot is closer to the localization label arranged in the environment, thereby improving the localization accuracy. This study did not deploy ultrasound tags in greenhouse scenarios. In future work, we will deploy this module in actual greenhouse scenarios as an input to the system for large-scale scene localization. Based on the error characteristics of ultrasound tags, trajectory inference algorithms, and SLAM positioning algorithms, the fusion of the three can achieve robust localization in complex environments. This is also another research work we are currently conducting.

For agricultural robots, real-time localization of crop rows and turns in large-scale greenhouse environments must be achieved. The proposed AF-PCP SLAM algorithm can accurately estimate the trajectory, as shown in **Figure 12A**. The maximum average RPE is 0.046 m. The proposed method was shown to have high robustness and accuracy in challenging agricultural environments, outperforming the current state-of-the-art approaches.

## 5 Conclusion

Aiming to address the problem that low-density canopy environments in greenhouses affect the robustness and localization accuracy of SLAM methods, in this paper, a spatial downsampling map construction and localization method based on the Cartographer framework is proposed, an adaptive filtering spatial point cloud projection algorithm is designed, and a greenhouse map construction and high-precision pose estimation system are developed. For greenhouse crop leaf area densities ranging from 2.945 m^2^/m^3^ to 5.301 m^2^/m^3^, the method proposed in this paper can accurately model the contours of sparse crop rows. Compared with that of the Cartographer algorithm, the map area of the AF-PCP SLAM algorithm for the crop row increased by 155.7%. The mean error and coefficient of variation for the crop row length were 0.019 m and 0.217%, respectively, which were respectively 77.9% and 87.5% less than those of the Cartographer algorithm. The average maximum void length was 0.124 m, which was 72.8% less than that of the Cartographer algorithm. Localization tests were carried out at speeds of 0.2 m/s, 0.4 m/s, and 0.6 m/s. The average relative localization errors of the actual motion trajectory and the real path were 0.026 m, 0.029 m, and 0.046 m, respectively, and the standard deviations were less than 0.06 m. Compared with those of the track deduction algorithm, the average localization error was reduced by 79.9%. These results show that the method can meet the requirements of map construction and localization in greenhouse environments. Thus, the proposed method is an effective approach for the autonomous operation of agricultural robots, providing a basis for localization and perception for efficient decision-making and safe operation of intelligent agricultural machinery and equipment in greenhouses.

## Data availability statement

The raw data supporting the conclusions of this article will be made available by the authors, without undue reservation.

## Author contributions

HT: Conceptualization, Data curation, Funding acquisition, Investigation, Methodology, Resources, Supervision, Validation, Visualization, Writing – original draft, Writing – review & editing. XZ: Conceptualization, Data curation, Investigation, Methodology, Software, Validation, Writing – review & editing. CZ: Conceptualization, Funding acquisition, Investigation, Methodology, Project administration, Resources, Supervision, Validation, Writing – original draft, Writing – review & editing. HF: Software, Visualization, Writing – review & editing. LC: Formal Analysis, Funding acquisition, Writing – review & editing. MY: Formal Analysis, Funding acquisition, Writing – review & editing.
